# SummonChimera infers integrated viral genomes with nucleotide precision from NGS data

**DOI:** 10.1186/s12859-014-0348-4

**Published:** 2014-10-22

**Authors:** Joshua P Katz, James M Pipas

**Affiliations:** Department of Biological Sciences, University of Pittsburgh, Pittsburgh, PA 15260 USA

**Keywords:** Integration, Virus, Cancer

## Abstract

**Background:**

Viral integration into a host genome is defined by two chimeric junctions that join viral and host DNA. Recently, computational tools have been developed that utilize NGS data to detect chimeric junctions. These methods identify individual viral-host junctions but do not associate chimeric pairs as an integration event. Without knowing the chimeric boundaries of an integration, its genetic content cannot be determined.

**Results:**

Summonchimera is a Perl program that associates chimera pairs to infer the complete viral genomic integration event to the nucleotide level within single or paired-end NGS data. SummonChimera integration prediction was verified on a set of single-end IonTorrent reads from a purified Salmonella bacterium with an integrated bacteriophage. Furthermore, SummonChimera predicted integrations from experimentally verified Hepatitis B Virus chimeras within a paired-end Whole Genome Sequencing hepatocellular carcinoma tumor database.

**Conclusions:**

SummonChimera identified all experimentally verified chimeras detected by current computational methods. Further, SummonChimera integration inference precisely predicted bacteriophage integration. The application of SummonChimera to cancer NGS accurately identifies deletion of host and viral sequence during integration. The precise nucleotide determination of an integration allows prediction of viral and cellular gene transcription patterns.

**Electronic supplementary material:**

The online version of this article (doi:10.1186/s12859-014-0348-4) contains supplementary material, which is available to authorized users.

## Background

Viral integration is a ubiquitous biological process where a virus inserts its genetic material into a host genome. Integration sometimes results in the entire viral genome being incorporated into the host genome, such as occurs with lysogenic bacteriophage or retroviruses [1]. In other cases, only a portion of the viral genome is integrated. For example, sub-genomic segments of Human Papilloma virus or Hepatitis B virus (HBV) genomes are sometimes integrated in cervical and hepatocellular cancers (HCC) respectively [2,3]. By identifying the lost and gained sub-genomic segments of host and virus, potential expression of viral and host genes can be estimated.

There is a growing amount of Next Generation sequencing (NGS) data available for cancer genomes [4-7]. These datasets allow for massive viral integration analysis in which single virus and host chimeras have been identified and compared [5-10]. Some experimental studies have identified the complete viral integration by PCR [11]. Current viral integration detection software programs [12-14] are designed to identify virus-host chimeras and report their genomic positions. However, the complete mapping of viral integrations requires the association of two chimeric sequences representing the two virus-host junctions present in each integration event. SummonChimera is a Perl program developed to detect chimeras from paired or single end NGS reads then associate two chimeras that describe the integration event. Mapping and association of both virus-host junctions allows the identification of viral and host sequences retained and lost during integration.

## Implementation

### Datasets

**Figure 1:**
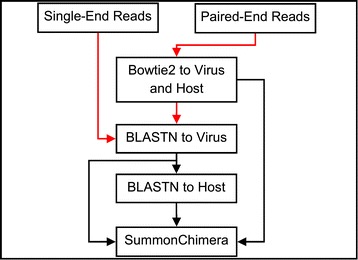
**Chimera detection pipeline.** Red arrows represent unaligned reads. Black arrows represent aligned reads and data. This figure outlines the standard computational subtraction pipeline used to generate SummonChimera input. Mapping of paired-end reads must occur to a database composed of both virus and humangenomes with Bowtie2. The BLASTN steps are ordered for optimal search time. Read counts at each step of the pipeline for each sample can be found in Additional file 1.

The single-end Ion Torrent sequencing reads of a purified *Salmonella enterica* culture, contained 2,295,084 reads with an average length of 132 residues, was obtained from Russell *et al.* [15] and is publicly available. No IRB approval is required for these studies. Hepatocellular carcinoma (HCC) paired-end Whole Genome Sequencing (WGS) reads were generated on an Illumina HiSeq 2000 sequencer platform by Sung *et al*. [7]. The reads were publicly available in the European Nucleotide Archive [ENA:ERP001196] (http://www.ebi.ac.uk/ena/data/view/ERP001196). Samples T198 and T268 from the HCC WGS study, with 50,176,914 and 54,352,036 paired-end reads respectively, were chosen for two reasons. First, subsets of chimeras were experimentally verified [7]. Second, these samples were used by Wang *et al.* [12] to compare current integration detection software [12-14]. PRINSEQ [16] quality filtering was run on the detected chimeric reads from each dataset to eliminate reads with an average quality less than 15. Sequencing sets were run through the alignment subtraction pipeline outlined in Figure 1 (see Additional file 1).

### Paired-end mapping

Default Bowtie2 [17] settings were used for mapping of discordant paired-end reads. For both HCC samples, a single Bowtie2 database was created with the UCSC hg19 human genome (http://hgdownload.soe.ucsc.edu/downloads.html) and the HBV genome [Genome:NC_003977] from the NCBI Genome database (http://www.ncbi.nlm.nih.gov/genome/). No mapping was run on the single end IonTorrent Salmonella dataset.

### BLASTN pipeline

The BLASTN [18] subtraction pipeline (Figure 1) was used for the identification of chimeric reads. In cases where the reads were mapped, all unmapped reads were extracted from the resulting BAM file with ‘samtools –f 4’. BLASTN parameters ‘-use_index true’, ‘–outfmt 6’, ‘-word_size 16’, and ‘-perc_identity 95’ are used to minimize output file size and computation time of each step while maximizing the number of detected chimeras. The input reads are first aligned to the virus database with BLASTN. Reads with a virus BLAST hit were then searched against the host database with BLASTN. HCC BLAST databases used the same reference sequences that were used during the mapping step: host hg19 and virus HBV. The Salmonella BLAST databases were composed of the virus genome Salmonella bacteriophage RE-2010 [Genome:NC_019488] and host genome *Salmonella enterica* [Genome:NC_011294] from the NCBI Genome database. BLAST databases and indices were also created with default parameters.

### SummonChimera (SC)

SC was written such that sequence processing is left to the users. BLAST and mapping are all done separate from SC and results are used as input. Figure 1 indicates the pipeline to be used for analysis. SC was run with default parameters using the BLAST output and SAM files as input.

SC’s first steps are to filter confounding results from the BLAST analysis. BLAST alignments are filtered based on user defined parameters to determine which alignments are suitable. From the remaining BLAST results, alignments with the highest bit score to virus and host are used to characterize the chimera. Chimeric reads with alignments having equivalent bit scores to multiple positions in virus or host are removed from analysis. All mapped virus-host discordant pairs identified in the SAM file are treated as a chimera.

**Figure 2:**
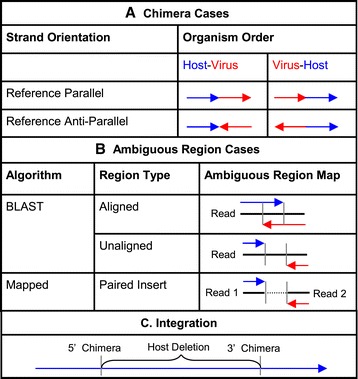
**Chimeric cases. (A)** There are four possible cases of chimeric reads. Arrows point from 5’ to 3’ on the reference strand of the host. Blue arrows represent host genome and red is virus. The two columns indicate organism order cases, which as illustrated is always determined by comparison to the host reference strand. There are two possible strand orientations which are determined by the orientation of the host and virus reference strand to each other. **(B)** Three ambiguous cases for chimeric reads are possible. BLASTN finds a single chimeric read and thus there is potential for overlap or void positions in the alignment. Mapped chimeras are two reads with an unknown nucleotide distance between them, as such their ambiguous region is defined as the closest two nucleotides mapped from virus and host. **(C)** Reference parallel and anti-parallel integrations are reported if they are within the user defined host deletion size.

Chimeras were categorized in three ways: strand orientation, organism order, and ambiguous region (Figure 2). Strand orientation indicates whether the virus has integrated its genome parallel or anti-parallel to the host genome. Parallelism was determined by the virus and host reference strand as listed in the NCBI Genome Genbank record. A parallel integration was defined as the viral reference strand covalently bonded to the host reference strand. Reference anti-parallel would be the virus non-reference strand linked to the host reference strand. Organism order is the 5’ to 3’ order of the organisms represented in the chimera based on the host reference strand. Integrations are inferred from strand orientation and organism order (Figure 2A). Finally, an ambiguous region (Figure 2B) is defined as the portion of the chimera that cannot be distinguished as virus or host. Chimeric reads were clustered based on these three characteristics. BLAST chimeras were clustered if they shared all three characteristics and occurred at the same host and virus position. Mapped and BLAST chimeras were clustered if they had equivalent organism order, strand orientation, and occurred within a user defined paired-end read insert size on both virus and host. Insert size is used for clustering chimeric reads because it is hypothetically the farthest possible nucleotide distance between a mapped chimeric read and the chimeric junction as determined by BLAST. The HCC dataset insert size was 500 nucleotides. Occasionally multiple BLAST chimera ambiguous regions were associated with a cluster. In these cases, the most frequent BLAST ambiguous region was reported.

Integration predictions were determined by the association of chimeras within the user defined nucleotide deletion size on the host genome (Figure 2C). Nucleotide deletion size is the maximum expected portion of the host genome removed during viral integration. Further, chimeras involved in the integration must have equivalent strand orientation and host flanking virus organism order. Figure 2A rows (Reference Parallel and Reference Anti-Parallel) illustrate the two cases of chimera pairs that represent integration events. All chimeras that are not paired are reported as orphan chimeras. The user determines whether a reported orphan chimera or integration is real based on the context of their study.

## Results

SC was tested on two different data sets, a Salmonella bacterium culture known to harbor an integrated bacteriophage and HBV integration in HCC. The Salmonella dataset was used to verify our integration inference method by comparison of SC results to a known integration of bacteriophage into a Salmonella bacterium [1,15]. Second, two HCC tumor samples were used to show the application of SC on cancer NGS data and its ability to identify and associate experimentally verified chimeras.

### Bacteriophage integration

In some types of lysogenic bacteriophage integration into the host occurs within the homologous core of attP and attB sites. AttP occurs at the single stranded 5’ and 3’ ends of the linear bacteriophage genome. When a bacteriophage genome enters the cell it circularizes and a double stranded attP is formed. The enzymatic integration occurs without loss to the bacteriophage or bacterial genome [1]. We tested SC on a bacterial strain known to harbor an integrated bacteriophage genome.

**Figure 3:**
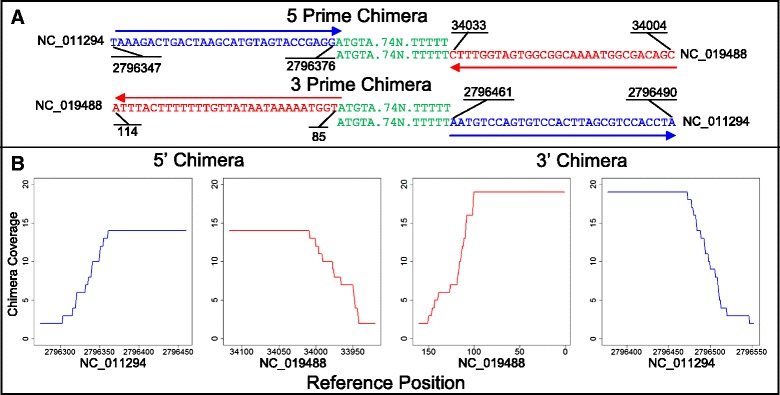
**SummonChimera prediction of a Salmonella bacteriophage Integration. (A)** These are segments taken from the aligned region of a 5’ and 3’ chimera. Blue is bacterial sequence [NCBI Genome: NC_011294], red is bacteriophage sequence [NCBI Genome:NC_019488], and green is homology to both. The numbers indicate the reference sequence nucleotide position. The ambiguous region (green) is 84 nucleotides long. The blue (host) and red (virus) arrows show the 5’ to 3’ direction of the host and virus reference strands. **(B)** The number of reads (depth of coverage) at each nucleotide position in the 5’ and 3’ chimeras for host and virus are shown. The ambiguous region of the chimera is counted twice (once for the host and virus positions) due to the unknown nature of where the integration occurs.

The genome of a Salmonella strain carrying a prophage was sequenced by Ion Torrent using single end reads [15]. Thus, only BLASTN alignments were utilized for SC input. SC identified a total of 69 chimeric reads and reported one integration with ten possible combinations of 5’ and 3’ ambiguous regions (see Additional file 2). Genome coverage of the 32 chimeric reads associated with the 5’ and 3’ ambiguous regions illustrates the integration prediction (Figure 3B). The remaining 37 reads (see Additional file 2) are individual chimeras and are likely artifacts of the sequencing process based on lack of chimeric junction coverage. PRINSEQ identified no chimeric reads with an average quality less than 15. The 5’ chimeras were divided between two ambiguous regions and the 3’ chimeric reads were divided into five ambiguous regions. Each ambiguous region had single nucleotide variations which likely resulted from sequencing error neighboring the chimeric junction. The integration with the most reads for the 5’ and 3’ chimera (8 and 11 reads respectively) was selected from SC output for further analysis. The selected integration showed that the nucleotide compositions of the 5’ and 3’ chimera ambiguous regions were the same. Further, the chimeras occurred at the 5’ and 3’ ends of the bacteriophage genome. Analysis of the bacterial indices indicated that the bacterial genome remained intact (Figure 3A). SC predicted the identical integration as was determined by WGS DNA sequencing [15].

### Hepatocellular carcinoma HBV integration

In contrast to integration by lysogenic bacteriophage, the integration of DNA tumor virus genomes occur at random positions with respect to both the viral and host genomes. For example, when HBV is integrated in HCC there is a loss of both viral and human DNA. Experimentally determined HBV integrations show no nucleotide specific human integration site [7,11,19].

**Figure 4:**
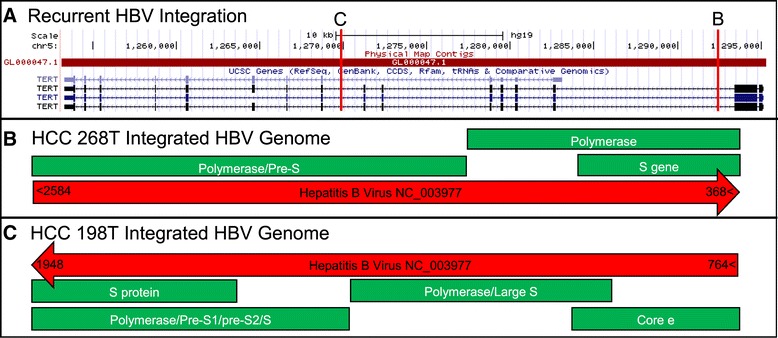
**SummonChimera detected recurrent HBV integration in HCC. (A)** The human chromosome 5 region with the hTERT gene from the UCSC Genome Browser [http://genome.ucsc.edu/] view of the hg19 is shown [20]. hTERT is a known recurrent HBV integration site. The red vertical lines indicate the positions of HBV integration, as predicted by SC, that are illustrated in B and C. **(B)** The T268 HCC sample HBV integration has been shown to be reference parallel. The red arrow points 5’ to 3’ on the virus reference strand and the numbers indicate the determined viral nucleotides included, keeping in mind HBVs circular genome. Green blocks represent the annotated gene coding regions within the integration. **(C)** The T198 HCC sample has an HBV integration that places the viral genes (green) in a transcription parallel orientation relative to hTERT.

To test the ability of SC to detect HBV integrations we examined two HCC datasets, T198 and T268, for which viral chimeras had been PCR verified [7]. This data has been used to verify other chimera detection software [12]. From these datasets SC identified 80 chimeric reads including all reported PCR verified chimeras (see Additional files 3 and 4). PRINSEQ was run on all detected chimeric reads and identified none with average quality less than 15. Additionally, SC identified a complete integration event in T198 and T268 by associating PCR verified chimeras (Figure 4).

Both integrations detected by SC occurred within the hTERT gene, a known recurrent integration site for HBV in liver cancer [5,7,11] (Figure 4A). Host positions of these integrations are similar but, their viral genetic content is different (Figure 4B and C). The integration in sample T268 (Figure 4B) is reference parallel and integrates the HBV non-coding strand into the coding strand of the hTERT gene. The HBV integration in sample T198 (Figure 4C) is reference anti-parallel, incorporating the HBV coding region into the hTERT coding strand. Thus while occurring in the same host gene these integrations have different biological implications. This result illustrates that the identification of the complete integration event by SC provides additional genetic information beyond current chimeric detection software.

## Discussion

Large NGS datasets containing genomic DNA or RNA sequences provides an opportunity to detect and examine viral integration events. The precise mapping of both virus-host junctions in individual tumor samples allows determination of the host gene in which integration occurs, the estimation of viral gene expression, the presence of virus-host chimeric mRNAs, and whether host and viral transcription have diverged from their known form.

SC was developed to map viral integration events from NGS. SC is designed to keep all analysis under user control and for usability with any NGS dataset. All chimeras and integrations detected by SC are output into a tab separated text document. Provided fields include ambiguous host and virus positions, aligned virus and host region, chimera strand orientation, organism order, host sequence name, and the nucleotide distance between the chimeras of a predicted integration. With the provided junctions for the 5’ and 3’ chimeras and strand orientation the lost portion of viral and host genome can be inferred.

SC predictions were validated against a variety of experimentally pertinent data. SC detected 100% of the verified chimeras from a HCC dataset and predicted two previously unknown integrations within the HCC genomes. Current reports suggest that an integration event may either activate or suppress neighboring host and viral genes [2,11]. Since SC determines the integrated viral genome and lost human genome, this information can be utilized to predict effects on host and viral transcripts.

NGS generates artificial chimeras that could be falsely interpreted as an integration by SC. Assuming artifact chimeras are generated at random the number of reads covering the same junction will be low relative to authentic chimeric junctions. We developed a model to predict the probability of false-positive integration calls by SC. The equation and calculations for the Salmonella and HCC datasets are supplied (see Additional file 5) [21]. Chimeric junctions and artifact chimeras are manually differentiated by read coverage. It is incumbent upon the user to determine the authenticity of chimeras and precision of called integrations based on the context of their experiment.

**Figure 5:**
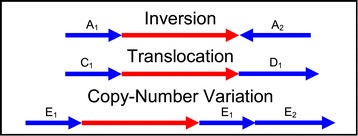
**SummonChimera undetectable integrations.** Viral integrations in cancer have been correlated with chromosomal inversion, translocation, and Copy-Number variation events. When integrations occur as illustrated, SummonChimera cannot predict the genetic content. Arrows point from 5’ to 3’ along the reference strand of each organism chromosome (host is blue and virus is red). Segment labels indicate a portion of the host genome. In the Inversion case one of the chimeras will be determined with opposite organism order and strand orientation, thus eluding integration prediction. In case of translocation, the integration can occur between two separate host chromosomes. The two chimeras will be detected on separate chromosomes and no integration would be predicted. Finally, the Copy-number Variation case, if the integration occurs between the duplicated segments the chimeras will be detected in the wrong chromosomal positions and the 3’ chimera will appear to be upstream of the 5’ chimera on the chromosome.

Recent studies of HPV integrations in cervical cancer have shown a positional correlation to genomic instability. These genomic instability events included copy-number variations, translocations, and inversions [6]. This genomic instability correlation implicates a potential problem for integration inference. When a viral integration occurs near any of these events (Figure 5), then the chimeric cases (Figure 2) in SC are not sufficient for integration prediction. Further work is required to include genomic instability mapping into SC in order to describe integrations within these regions.

## Conclusions

Current viral integration software only detects and reports the position of individual chimeras. SC reports chimera organism order, strand orientation and associates appropriate chimeras to accurately infer the genetic content of viral integrations (see Additional file 6). With the identification of the strand orientation and lost portion of host and viral genome from an integration event, the molecular and genetic consequences of integration can be predicted.

## Availability and requirements

**Project Name:** SummonChimera

**Project home page:**http://pipaslab.webfactional.com/wp/wp-content/uploads/2014/01/SummonChimera.tar.gz

**Operating System(s):** Linux, Mac OS X, Windows

**Programming language:** Perl

**Other requirements:** BLAST + 2.2.28 and Bowtie2 2.1.0

**License:** GNU

**Restrictions for use by non-academics:** None

## Additional files

Additional file 1:
**Read Counts for Figure** 1 **Computational Subtraction Pipeline.** Description of Dataset: Read counts for aligned and unaligned at each step of the pipeline. Additional file 2:
**SummonChimera Output for Salmonella.** Description of Dataset: All reported integrations and orphan chimeras for the Salmonella data. Additional file 3:
**SummonChimera Output for Hepatocellular Carcinoma T198.** Description of Dataset: All reported integrations and orphan chimeras for the T198 data. Additional file 4:
**SummonChimera Output for Hepatocellular Carcinoma T268.** Description of Dataset: All reported integrations and orphan chimeras for the T268 data. Additional file 5:
**Calculations of False-Positive Integration Calls by SummonChimera.** Description of Dataset: Contains developed equation and calculations for all three datasets. Additional file 6:
**SummonChimera.** Description of Dataset: Contains SummonChimera source code and test data.

## Abbreviations

HPV: Human Papilloma virus
HBV: Hepatitis B Virus
SC: SummonChimera
HCC: Hepatocellular carcinoma
WGS: Whole genome sequencing
NGS: Next generation sequencing
