# Supplementary material for: SummonChimera infers integrated viral genomes with nucleotide precision from NGS data
Source: BMC Bioinformatics. 2014 Oct 22;15(1):348. doi: 10.1186/s12859-014-0348-4 (PMC4210586; doi:10.1186/s12859-014-0348-4)
Supplement: Additional file 5: — Calculations of False-Positive Integration Calls by SummonChimera. Description of Dataset: Contains developed equation and calculations for all three datasets. [file 12859_2014_348_MOESM5_ESM.docx]

$${P=\left( \frac{\left( H_{r}+V_{r} \right)*\frac{C}{R}}{H_{g}-D} \right)}^{2}*\frac{1}{8}$$

P – Probability of false-positive integration

H_r_ – Number of host reads

V_r_ – Number of virus reads

C/R – Artificial chimeric read generation proportion for Multiple Displacement Amplification [21]

H_g_ – Number of expected host nucleotides

D – Size of expected host deletion during integration

1/8 – The probability both chimeras will be in the proper orientation

False-Positive Integration Probability Calculations

Salmonella Dataset

H_r_ = 1,920,721 V_r_ = 14,546 C/R = 4.5e-3 H_g_ = 4,685,848 D = 0

P = 4.3e-7

HCC Dataset T198

H_r_ + V_r_ = 96,911,230 C/R = 4.5e-3 H_g_ = 2,897,310,462 D = 500

P = 2.8e-9

HCC Dataset T268

H_r_ + V_r_ = 105,628,475 C/R = 4.5e-3 H_g_ = 2,897,310,462 D = 500

P = 3.4e-9
